# Investigation of the effects of Bipolar Radiofrequency Energy on the Structural Morphology of Dental Plaque

**DOI:** 10.1055/s-0043-1764427

**Published:** 2023-05-12

**Authors:** Bennett T. Amaechi, Sahar Mohseni, Andrew M. Dillow, Parveez Ahamed Abdul Azees, Fatemeh Movaghari Pour, Yuko Kataoka, Maria Camila Restrepo

**Affiliations:** 1 Department of Comprehensive Dentistry, School of Dentistry, University of Texas Health Science Center at San Antonio, San Antonio, Texas, United States; 2 School of Dentistry, CES University, Medellin, Colombia

**Keywords:** dental plaque, power toothbrush, radiofrequency, toothbrushing, ToothWave, bacterial viability

## Abstract

**Objectives** To investigate the effects of radiofrequency (RF) energy, applied through a power toothbrush, on the structural morphology of dental plaque and its bacteria components. Previous studies showed that a toothbrush powered by RF (ToothWave) effectively reduces extrinsic tooth stains, plaque, and calculus. However, the mechanism by which it reduces dental plaque deposits is not fully established.

**Materials and Methods** Multispecies plaques at sampling time points of 24, 48, and 72 hours were treated with the application of RF using ToothWave with the toothbrush bristles 1 mm above the plaque surface. Groups that underwent the same protocol but without RF treatment served as paired controls. Confocal laser scanning microscope (CLSM) was used to determine cell viability at each time point. Plaque morphology and bacteria ultrastructure were viewed using scanning electron microscope (SEM) and transmission electron microscope (TEM), respectively.

**Statistical Analysis** Data were analyzed statistically using analysis of variance (ANOVA) and Bonferroni post-tests.

**Results** At each time, RF treatment significantly ( *p* < 0.05) reduced the viable cells in plaque and caused a substantial disruption of plaque morphology, while the untreated plaque had intact morphology. Cells in treated plaques showed disrupted cell walls, cytoplasmic material, huge vacuoles, and heterogeneity in electron density, while these organelles remained intact in untreated plaques.

**Conclusion** The application of RF via a power toothbrush can disrupt plaque morphology and kill bacteria. These effects were enhanced by the combined application of RF and toothpaste.

## Introduction

The oral environment is exposed to permanent colonization by a variety of microorganisms that are, in normal health circumstances, in balance with the immune system of the host. Within that environment, the hard dental surfaces are prone to adhesion of a thin biofilm formed by a highly structured and organized bacterial community. 1 When it is not routinely eliminated, this biofilm, which constitutes dental plaque, can progressively accumulate supra- and subgingivally and become mineralized, forming calculus. 2 These bacterial deposits stimulate a local host response manifested by gingival inflammation and alter the pH of the saliva and biofilm on tooth surfaces, causing demineralization of dental tissues. 3 Therefore, dental plaque is considered the leading etiological factor of gingivitis and dental caries. 3 Gingivitis is a reversible phenomenon, but if it is left untreated, the inflammation can propagate to the subjacent periodontium, causing permanent damage to the dental supporting structures and inducing periodontitis. 4 Oral hygiene tools used at home do not currently provide complete elimination of dental plaque from the oral cavity. 5 6 However, routine and efficient toothbrushing can disrupt the structural integrity of the biofilm, and restore the normal oral microbial population, thus significantly reducing the destructive effects of the biofilm, reversing gingival inflammation, and maintaining good oral health. 5 7 8 This efficiency is contingent upon the subject's manual dexterity, the type of toothbrush used, and the brushing technique, time, and frequency. 8

In the market since the early 1960s, powered toothbrushes have been taking up an increasing share of toothbrush sales, becoming more widely used as an alternative to manual toothbrushes. 9 The development of powered toothbrushes aimed to improve many of the parameters mentioned earlier to achieve a more efficacious brushing routine. Powered toothbrushes can clean dental surfaces through a direct physical action of the bristles, just like manual toothbrushes, and through hydrodynamic effects associated with the bristles' motion and speed superior to manual toothbrushes. 10 11 12 Thus, the unique characteristics of these powered toothbrushes have established their efficacy in reducing stains and deposits from dental surfaces. 13 14 15 Studies have also demonstrated that powered toothbrushes are as safe to use as manual toothbrushes with no adverse effects on the soft and hard oral tissues when regular forces are applied during brushing. 16 17 18 19 20 Currently many types of powered toothbrushes are available. They can be categorized according to the kind of movement of the bristles (vibrational, oscillation-rotational, or circular); speed of movement (sonic or ultrasonic); type of electric current, direct or alternating current (electronic or ionic toothbrushes) 9 21 ; and whether it is powered by a unique technology (radiofrequency [RF] energy). 14

RF technology has made its way into the medical field through various applications, one of the latest being its use in the novel powered toothbrush, ToothWave (Home Skinovations, Israel). This toothbrush uses RF energy, which is a low-power alternating current. This current oscillates from 3 kHz to 300 GHz and circulates between two electrodes and over a silicon barrier. Previous studies have shown that this toothbrush using RF energy significantly reduces extrinsic tooth stains, plaque, calculus, and subsequent gingivitis. 14 15 22 It is an effective tool that is easy to incorporate into one's oral hygiene routine at home while being nonabrasive and well-tolerated. 14 23 As for its mechanism of action, it is hypothesized that during toothbrushing, the alternating RF current propels the toothpaste's active molecules onto the tooth surface, compromising the adhesion of bacterial deposits and stains to the enamel surface. However, no studies have established the mechanism by which RF energy causes a reduction in dental plaque deposits. Therefore, the aim of this *in vitro* study was to investigate the effects of a bipolar RF (3 W, 3 MHz) energy applied through a powered toothbrush on the structural morphology (chain formation) of periodontal disease-causing dental plaque and the ultrastructure (cell wall and cell components) of the bacteria components. We hypothesized that the application of 3-MHz bipolar RF delivered via a powered toothbrush RF electrode after biofilm formation would cause structural disintegration of dental plaque and disruption of the bacterial cellular component.

## Materials and Methods

### Bacterial Strains and Growth Conditions

Three red complex bacteria ( *Porphyromonas gingivalis* ATCC 33277, *Treponema denticola* ATCC 35404, and *Tannerella forsythia* ATCC 43037), known to be the major components of dental plaque responsible for causing periodontal diseases, 3 were used in this study. All cultures were grown at 37°C in an anaerobic incubator containing a gaseous mix of 90% N _2_ , 5% H _2_ , and 5% CO _2_ for 2 to 4 days. *P. gingivalis* was grown in tryptic soy broth supplemented with hemin (5 μg/mL) and cysteine (0.5 g/L), *T. denticola* in modified NOS medium, and *T. forsythia* in tryptic soy broth supplemented with 0.3% yeast extract, vitamin K (0.4 mg/mL), and N-acetylmuramic acid (NAM; 10 mg/mL; Sigma Aldrich, St. Louis, Missouri, United States). After growth, a loop full of individual colonies was transferred from agar plates to tubes containing oral bacteria growth medium (OBGM) containing brain heart infusion (12.5 g/L), tryptic soy broth (10 g/L), yeast extract (7.5 g/L), sodium thioglycolate (0.5 g/L), asparagine (0.25 g/L) D-glucose (2 g/L), ascorbic acid (2 g/L), sodium pyruvate (1 g/L), sodium bicarbonate (2 g/L), L-cysteine (1 g/L), ammonium sulfate (2 g/L), thiamine pyrophosphate (6 mg/L), heat-inactivated rabbit serum (5% vol/vol), hemin (5 mg/L), menadione (1 mg/L), NAM (10 mg/mL), and a volatile fatty acid mix (0.5% vol/vol). Each tube was incubated at 37°C in an anaerobic chamber (85% N _2_ , 10% H _2_ , 5% CO _2_ ) for 48 hours to attain active exponential growth phase culture to prepare an active exponential phase multispecies culture.

### Experimental Grouping

A total of 360 acrylic disks were fabricated, each measuring ∼6 mm diameter × 0.3 mm thickness, serving as the substrate on which the biofilm was grown. The disks were assigned to the following six experimental groups (45 disks/group) consisting of 3 control groups not treated with RF energy (A, B, C) and 3 test groups (D, E, F) treated with RF energy: (A) bacterial growth medium only, (B) toothpaste slurry (TS) only, (C) centrifuged toothpaste slurry (CTS) only, (D) RF treatment in the growth medium, (E) RF and TS treatment, and (F) RF and CTS treatment.

### Biofilm Formation in Microbial Plaque Model

**Fig. 1:**
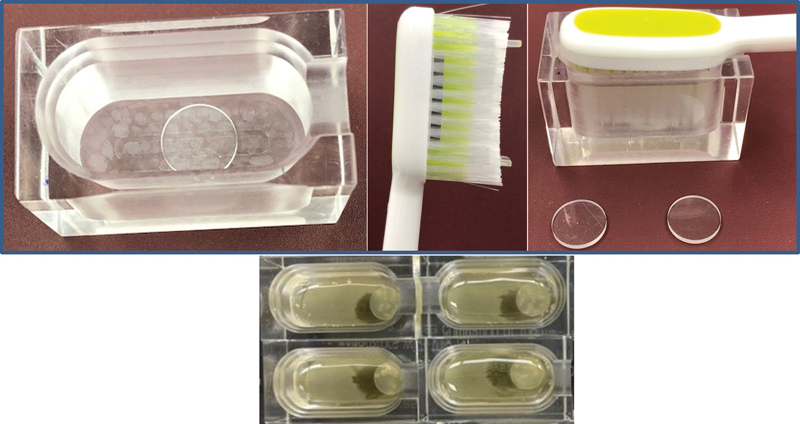
Setup of the microbial model (MM) serving as the culture bath as well as treatment bath.

The microbial model (MM) is shown in Fig. 1 . The model consists of a compact-sized, custom-made rectangular acrylic culture bath to accommodate just the toothbrush head and its bristles. A thin circular acrylic disk served as the substrate on which the dental plaque was grown and placed on the floor of the well. Attached to the toothbrush head at the two ends of the bristles are two acrylic stubs positioned to give the bristles 1-mm clearance from the circular disk on the floor of the well. The compact design ensured that when the toothbrush was positioned inside the well, the toothpaste was only accommodated within the bristles and the space between the bristles and the floor of the well, thus ensuring the minimum amount of toothpaste was used. The liquid volume must be as low as possible because a high liquid volume will dilute the charged molecules and prevent the formation of a high concentration of charged molecules on the plaque surface.

Before the start of the study, all components were aseptically set up, following sterilization in an autoclave for 20 minutes at 121°C (15-lb pressure). The growth medium used in this system was a 2% sucrose-supplemented OBGM, and the pH was adjusted to 7.2. The entire assembly was housed inside an anaerobic chamber containing a gaseous mix of 85% nitrogen (N _2_ ), 10% hydrogen (H _2_ ), and 5% carbon dioxide (CO _2_ ) and maintained at a constant physiological temperature of 37°C.

To aid bacterial adhesion for biofilm formation, acquired salivary pellicles were formed on the enamel disks by a 30-minute immersion in filtered human saliva, with mild agitation at 70 rpm at 37°C. To initiate bacteria biofilm formation on the acrylic disks, each culture bath bearing a disk was filled with 20 mL of OBGM inoculated with the multispecies inoculum of the three red complex bacteria at medium-to-inoculum ratio of 10:1. Then, each of the culture baths was incubated at 37°C in an anaerobic chamber (85% N _2_ , 10% H _2_ , and 5% CO _2_ ) for 4 hours (adhesion phase). Following the 4-hour adhesion phase, the OBGM was replaced with bacteria-free fresh sucrose (2%) supplemented OBGM and incubated anaerobically (85% N _2_ , 10% H _2_ , and 5% CO _2_ ) for 24 hours at 37°C (biofilm maturation phase). Following this maturation phase (24-hour biofilm growth), each biofilm-bearing acrylic disk in its respective group was treated as follows:

- **Group A:** Neither RF device nor toothpaste was used. Biofilm-bearing disks were exposed only to the growth medium.
- **Group B:** The growth medium was replaced with a slurry of standard fluoride toothpaste (sodium monofluorophoshate, Colgate-Palmolive, New York, NY, United States) for 4 seconds, the estimated time for brushing each tooth surface *in vivo* , and then the slurry was rinsed off with sterile phosphate buffer saline (PBS). The TS was prepared by mixing one part toothpaste and three parts distilled water using a laboratory stand-mixer until homogenous.
- **Group C:** A slurry of standard fluoride toothpaste was prepared as described earlier. Then enough quantity of the slurry was centrifuged for 10 minutes at 10,000 rpm (4,000 g) at 7°C, and the supernatant fluid (CTS) was used for the treatment of this group. The enamel disks were covered with CTS for 4 seconds, and then the CTS was rinsed off with PBS.
- **Group D:** Using a home-use device, Silk'n power toothbrush Model H7001 (ToothWave), each biofilm-bearing disk was treated with a 3-MHz bipolar RF current for 4 seconds at a 1-mm distance between the surface of the toothbrush bristles and the biofilm-bearing disk. The toothbrush was activated on mode 4 (RF only, no vibration). The disks were treated inside the OBGM with the power toothbrush inserted into the medium.
- **Group E:** The culture bath containing the biofilm-bearing disk was filled with TS, then the power toothbrush was inserted into the culture bath. The disk was immediately treated with a 3-MHz bipolar RF current for 4 seconds as described for group D. After treatment, the TS was rinsed off with PBS.
- **Group F:** The culture bath containing the biofilm-bearing disk was filled with CTS, then the power toothbrush was inserted into the culture bath. The disk was immediately treated with a 3-MHz bipolar RF current for 4 seconds as described for group D. After treatment, the CTS was rinsed off with PBS.

Following each treatment, the OBGM was replaced with a fresh, bacteria-free OBGM. Over the next 2 days, the OBGM was replaced with an equal volume of fresh bacteria-free OBGM every 12 hours, and the respective treatment for each group was repeated every 24, 48, and 72 hours of biofilm formation. However, every 24 hours following treatment, 15 disks were aseptically harvested from each group and rinsed thoroughly in sterile PBS to remove planktonic cells and traces of OBGM, TS, and CTS.

Of the 15 disks from each group, 5 were used for confocal laser scanning microscope (CLSM) examination to determine the live-to-dead bacteria ratio, 5 for scanning electron microscope (SEM) examination to determine the effect on dental plaque morphology, and 5 for transmission electron microscope (TEM) examination to determine the effect on the bacteria's ultrastructure. These examinations were performed for each time point (24, 48, 72 hours).

## Structural Analysis of the Dental Plaque

### SEM Examination of the Effect on Dental Plaque Morphology

The biofilm-bearing disks were fixed for 2 hours in 2.5% glutaraldehyde solution at 4°C. After fixation, the disks were rinsed gently in 0.1 M phosphate buffer (pH 7.4; 3 times, 2 minutes each) and then placed in 1% Zetterquist's osmium tetroxide (OsO _4_ ) for 60 minutes. Afterward, the biofilms were rinsed with 0.1 M phosphate buffer (1 time, 2 minutes) and then in distilled water (2 times, 2 minutes each) to avoid contamination by insufficient osmium removal. Subsequently, the samples were dehydrated in an increasing series of ethanol dilutions (35, 50, 75, 2 × 90, and 2 × 100%) for 30 minutes each time in each solution. Then samples were immersed in hexamethyldisilazane (HMDS; Polysciences Inc., Warrington, Pennsylvania, United States) for 1.5 hours, and, finally, HMDS was removed. Biofilm surfaces were airdried in a desiccator for 12 hours. Each sample was coated with gold sputter after critical point drying and mounted on a glass slide. Finally, the biofilms were examined with an SEM (JCM-5700; JEOL, Tokyo, Japan) using the secondary electron emission mode with accelerating voltages of 1, 5, 10, and 20 kV and ×1,500 and ×5,000 magnifications.

### TEM Examination of the Effect on Bacteria Ultrastructure

The ultrastructure of the bacteria in the biofilm was examined by TEM analysis. Immediately upon harvesting and following rinsing with PBS, the biofilm-bearing disks were dropped into 1.5% paraformaldehyde and 2.5% glutaraldehyde fixative for 2 hours. Disks were postfixed in 2% osmium tetroxide for 2 hours, dehydrated in ethanol, and embedded in Araldite M (Merck, Darmstadt, Germany). The routinely performed embedding process of organic material for TEM examination was designed to transfer dehydrated specimens, step by step, from a pure propylene oxide solution to the embedding medium of Araldite M by way of intermediate Araldite–propylene oxide mixtures. In this process, the highly volatile, lipophilic solvent propylene oxide reduced the viscosity of the Araldite mixture and ensured a complete wetting and saturation of the structures to be embedded. The time for hardening lasted 48 hours at 65°C. After embedding, the biofilm on the disks was decalcified in 4% ethylenediaminetetraacetic acid (EDTA) at pH 7.2 and re-embedded. Ultrathin sections were cut from all biofilm-bearing disks on an Ultracut E ultramicrotome (Reichert, Benzheim, Germany) equipped with a Micro Star 55-degree diamond knife (Mikrotechnik, Benzheim, Germany) specially designed for sectioning materials. Serial ultrathin sections were mounted on Pioloform-F (Wacker-Chemie, Munich, Germany) coated copper grids, contrasted with uranyl acetate and lead citrate, and examined in a TEM 201 (Philips, Eindhoven, The Netherlands) at 80 kV. Representative micrographs were obtained at a magnification of ×30,000.

### CLSM Examination of the Effect on the Viability of the Bacteria in Biofilm

To use the CLSM to assess the plaque bacteria viability on the surface of each disk, the biofilm was stained with the L7012 Live/Dead BacLight bacterial viability kit (Molecular Probes, Life Technologies, Carlsbad, California, United States). The biofilm was stained by incubation in 50 μL of a dye solution for 30 minutes in the dark, at room temperature. The dye solution was prepared by dissolving 5 μL of SYTO and 5 μL of propidium iodide (PI) dye in 10 μL of DMSO, and then 980 μL of NaCl buffer (0.89%) was added to the dye solution to make up the volume of 1 mL. The dye solution was kept frozen until use. After staining, the excess stain was rinsed with sterile saline to avoid noise during the acquisition of the image under CLSM. After washing, the biofilm was immediately examined using an Olympus FLUOVIEW FV 1000 confocal scanning laser microscope with an FV-10 ASW system (Olympus Corporation, Tokyo, Japan) at excitation wavelengths of 485 (SYTO-Green Signals) and 543 nm (PI-Red Signals) with a 20X oil immersion lens. Images were obtained with the software FV10-ASW 4.0 Viewer (Olympus Corporation) at five random positions of the surfaces within the central periphery of the disk under the toothbrush, and a stack of maximum slices (1 μm thick each) was scanned. Finally, the Z Stack (surface topography and three-dimensional architecture) analysis was performed with FV10-ASW 4.0 Viewer (Olympus Corporation) to evaluate the number of live and dead bacteria at each location, and the average number for each disk was calculated.

### Statistical Analysis

The following examinations were performed with the data and the images. Using the SEM micrographs, the structural morphology of the plaque biofilm in all groups was evaluated and compared among the groups to identify and describe any effects of the RF treatment on the morphology of the biofilm at each assessment time point (24, 48, and 72 hours). Also, the effects of RF on biofilm morphology were confirmed by comparing the morphology of the biofilm in paired RF-treated versus non-RF-treated similar groups, that is, by comparing A versus D, B versus E, and C versus F at each sampling time point (24, 48, and 72 hours). Similarly, using the TEM micrographs, the effects of RF on the ultrastructure of the bacteria (cell wall and its components) were examined and compared between similar RF-treated and non-RF-treated groups (A vs. D, B vs. E, and C vs. F) at each sampling time point (24, 48, and 72 hours). Using the results from the CSLM, the effects of RF on the bacteria viability in each group were determined by comparing the percentage of viable cells between similar RF-treated and non-RF-treated groups (A vs. D, B vs. E, and C vs. F) at each sampling time point (24, 48, and 72 hours) using the two-way analysis of variance (ANOVA) with Bonferroni post-tests.

The primary hypothesis was that the application of 3 -MHz bipolar RF delivered via a power toothbrush electrode during and after biofilm formation would cause structural disintegration of dental plaque, disruption of the bacterial cellular component, and reduction of the number of live bacteria within the plaque. At the same time, the non-RF-treated control groups would exhibit none of these effects. This was tested statistically by STATA software version 17.0 (Stata Corp LP, United States) at a significance level of 5%. All pairwise contrasts (RF-treated vs. non-RF treated and between time points) were tested with ANOVA, followed by Tukey's honestly significant difference (HSD) test for multiple hypothesis adjustment.

## Results

### Confocal Laser Scanning Microscope Analysis

**Fig. 2:**
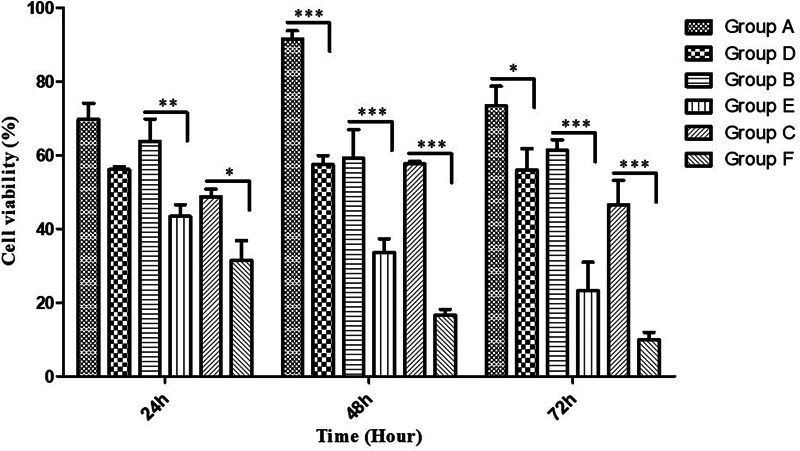
Combined graph for the three experimental days (24, 48, and 72 hours). Two-way ANOVA with Bonferroni post-tests was performed using GraphPad prism version 6.04, and all *p* values are two-sided and considered significant at *p* < 0.05. (* *p* < 0.05, ** *p* < 0.01, *** *p* < 0.001).

**Fig. 3:**
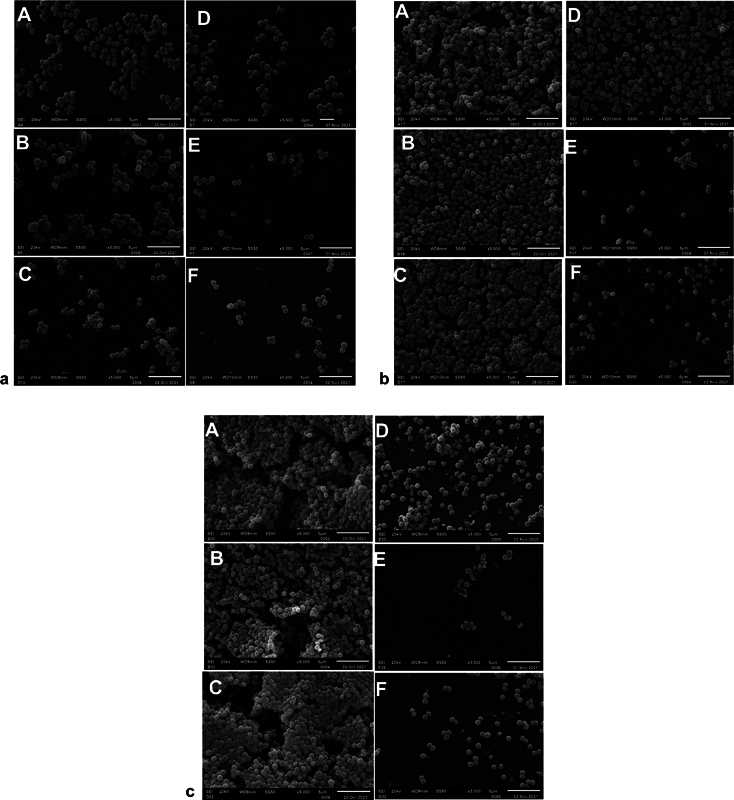
( **a** ) Scanning electron microscope images of 24-hour biofilm treated ( **D–F** ) and untreated ( **A–C** ) with radiofrequency (RF) energy and paired groups underwent a similar protocol. ( **b** ) Scanning electron microscope images of 48-hour biofilm treated ( **D–F** ) and untreated ( **A–C** ) with RF and paired groups underwent a similar protocol. ( **c** ) Scanning electron microscope images of 72-hour biofilm treated ( **D–F** ) and untreated ( **A–C** ) with RF and paired groups underwent a similar protocol.

In Fig. 2 , significant differences were observed in cell viability between the RF-treated and non-RF-treated groups. Overall, the biofilm treated with RF (groups D–F) showed a significant ( *p* < 0.5) reduction in the percentage of viable bacteria cells when compared with the untreated biofilm (A–C) that underwent a similar protocol. Without the toothpaste, this effect was not statistically significant at the 24-hour measurement period but became significant as the thickness of the biofilm increased at 48 and 72 hours. However, with toothpaste, the effect was significant ( *p* < 0.05) at all time points. At each time point, there was a significant ( *p* < 0.05) reduction in the percentage of viable bacteria cells with CTS than with TS, with or without RF treatment, except at 48 hours within the non-RF-treated groups ( Fig. 3 ).

### Scanning Electron Microscope Analysis

The SEM micrographs ( Fig. 3 ) of the non-RF-treated groups (A–C) showed a well-formed biofilm with intact structural colonization at each time point (24, 48, and 72 hours). However, in the samples treated with RF, the biofilm morphology (architecture) was substantially disrupted at each growth period. The biofilm morphology showed only a little difference among the three biofilm growth periods (24, 48, and 72 hours) in groups (B–D) treated with either toothpaste or RF, unlike the groups treated with combined RF and toothpaste (groups E and F). Basically, the SEM result showed that toothpaste enhanced the effectiveness of the RF against dental plaque.

### Transmission Electron Microscope Analysis

**Fig. 4:**
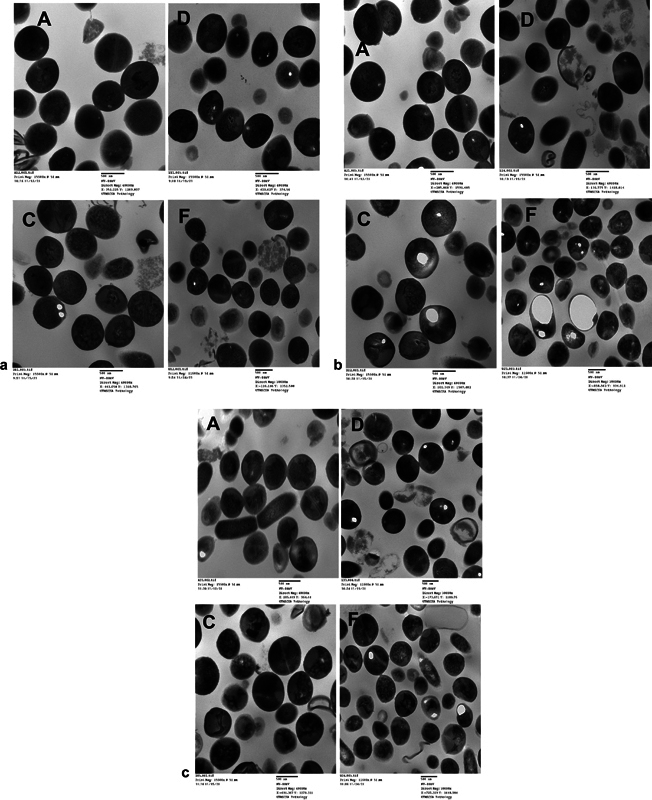
( **a** ) Transmission electron microscope images of bacteria in 24-hour multispecies biofilm treated ( **D,F** ) and untreated ( **A,C** ) with radiofrequency (RF) energy. ( **b** ) Transmission electron microscope images of bacteria in 48-hour multispecies biofilm treated ( **D,F** ) and untreated ( **A,C** ) with RF energy. ( **c** ) Transmission electron microscope images of bacteria in 72-hour multispecies biofilm treated ( **D,F** ) and untreated ( **A,C** ) with RF energy.

The TEM micrographs ( Fig. 4 ) showed bacteria in the biofilm grown with neither toothpaste nor RF treatment (group A) to have a typical shape, intact peritrichous flagella, peptidoglycan layer, and cytoplasmic membrane. In groups treated with either CTS (C) or RF (D), meager differences were observed, like lysed cells with broken walls and membranes and huge vacuoles. On the other hand, the bacteria in the biofilm (group F) treated with combined CTS and RF demonstrated morphological changes, including compromised cell wall and reduction and unevenness in electron density in the cytoplasm with the appearance of “ghost cells.” This again indicates more effectiveness with combined toothpaste and RF treatment.

## Discussions

The efficiency of manual toothbrushing to significantly eliminate plaque biofilm to maintain good oral health is contingent upon the individuals' manual dexterity, the type of toothbrush used, and the brushing technique, time, and frequency. 8 This has led to varieties of powered toothbrushes becoming more widely used as an alternative to manual toothbrushes, to provide more efficacious brushing than manual toothbrushes. 9 Among these powered toothbrushes is the ToothWave (Home Skinovations, Israel), which uniquely uses bipolar RF energy for its effectiveness in reducing exogenous deposits on tooth surfaces. Previous studies have shown that this RF-utilizing toothbrush significantly reduces extrinsic tooth stains, plaque, and calculus. 14 15 22 However, no studies have established the mechanism by which the RF energy causes the reduction in dental plaque deposits. Thus, the present study investigated the effects of bipolar RF energy applied through a power toothbrush on the dental plaque's structural morphology and the bacteria's ultrastructure. In the current study, to ensure that it is only RF current affecting the changes in the biofilm, both the mechanical vibration and the oscillating rotatory movements of the RF toothbrush were excluded by treating each biofilm at a 1-mm distance between the surface of the toothbrush bristles and the biofilm surface ( Fig. 1 ). The toothbrush was activated on mode 4 (RF only, no vibration). The study demonstrated, at every plaque thickness (24, 48, and 72 hours), a substantial disruption of the plaque architecture (chain formation) in the biofilms treated with RF, while the biofilms not treated with RF energy showed well-formed biofilm with intact structural morphology ( Fig. 3 ). This effect was observed to be grossly enhanced when RF treatment was combined with toothpaste (TS or CTS) treatment but less in the biofilm treated with either RF or toothpaste only at every plaque thickness ( Fig. 3 ). This result confirmed our hypothesis that the application of 3-MHz bipolar RF, delivered via a power toothbrush RF electrode after biofilm formation, would cause structural disintegration of dental plaque. The RF energy in ToothWave is a low-power alternating electrical current (AC), which streams back and forth between two electrodes and over a silicon barrier, providing a localized effect that is limited to the surface of the substrate on which the plaque is formed. This process is based on the principle of polarity, which states that every element in nature has a positive or negative charge. 24 The plaque disintegration effect observed in the present study, even at 3-day plaque thickness (matured plaque), can be attributed to the hypothesized mechanism of action of the ToothWave toothbrush, which involves a process in which the alternating current from the RF technology brings charged molecules that originate from the toothpaste to the dental plaque, to destabilize the electrostatic bonds between the individual bacterial cells and between the substrate on which the plaque is formed and the plaque that was attached firmly to the substrate. The high frequency of the AC set by the RF parameters allows for safely increasing the electrical power as opposed to direct current, thus achieving significant results. 25 Moreover, the RF current tends to flow along the surfaces of electrical conductors, known as the “skin effect,” 26 and thus directs the current toward the plaque surface. The electromechanical silicon barrier, located between the ToothWave electrodes, additionally contributes to its increased efficacy. The enhanced efficacy in disintegrating dental plaque observed with the introduction of toothpaste (TS or CTS) supported our suggested mechanism of action that the electrically charged toothpaste ingredients take part in the process that occurs in the biofilm. Toothpaste is a water-based complex mixture of abrasives and surfactants, humectants, binders, and other active ingredients. As such, it contains charged molecular compounds that act as electrolytes in the medium once the RF is activated, carry the charges within the plaque, and achieve the desired effect. The fact that the effect of the RF energy was observed at varying plaque thicknesses, even at 3-day plaque thickness, indicated that the effect of the RF energy was not limited to the surface of the plaque but penetrated deep into the entire thickness of a matured plaque.

The observed action of RF energy on the plaque in the present study is strongly believed to be responsible for the report of a previous comparative 6-week study that examined the effect of an RF-utilizing toothbrush on calculus, plaque, and gingival inflammation and reported a statistically more significant reduction in plaque and gingivitis in the test group that used RF toothbrush compared with the control group that used sonic vibrating toothbrush. 15 It is believed that the process described earlier by which RF energy disintegrates plaque architecture would apply to any exogenous materials (e.g., stains) that are firmly attached to a substrate layer, such as tooth surface, when an RF-utilizing toothbrush is used on such surface. For this reason, the result of the present study can be presumed to be responsible for the observation in another previous study that investigated the effect of this RF-utilizing toothbrush on stain deposits on the tooth surface and reported a more significant reduction in extrinsic dental stains with RF-utilizing toothbrush compared with the control sonic vibratory toothbrush. 14 These previous studies and the present study further support the beneficial effects of this unique technological feature, which utilizes RF energy that streams on the tooth surface during brushing.

We further visualized the established and treated biofilms with TEM to examine the ultrastructure of individual bacteria. TEM is a standard histology technique for viewing the ultrastructure of bacteria cells. It enables the cellular structures that allow the cell to function properly within its specific environment to be examined at an ultrastructural level. 27 TEM analysis of the dental plaque showed the bacteria in the biofilm grown with neither RF treatment nor toothpaste treatment to have all the typical structures and characteristics of live bacteria. At the same time, the bacteria components of the biofilm treated with either CTS or RF alone displayed all characteristics of lysed cells, such as broken walls and membranes and huge vacuoles ( Fig. 4 ). Again, this effect was more pronounced with combined CTS and RF in which the bacterial cells demonstrated more morphological changes, including compromised cell wall and reduction and unevenness in electron density in the cytoplasm with the appearance of “ghost cells” ( Fig. 4 ). This demonstrated that the cells' inner structure seemed strikingly affected by RF treatment. This observation can be attributed to the long-established effect of RF energy. RF is a type of dielectric heating that has the potential for uniform and rapid heating of samples through ionic conduction. 28 In the presence of an alternating electric field induced by RF, the displacement of ions with opposite charges leads to an increase in the kinetic energy of the molecules and, thus, to the rise in the temperature of the samples, in this case, the plaque biofilm. The DNA of microorganisms absorbs the heat generated inside the biofilm because of RF radiation, which subsequently changes their physical structure and reduces function. 29 For this reason, the RF has the capacity to lyse the bacterial cell wall and degenerate the cellular structures, with the charged molecules from the toothpaste being contributing factors. Furthermore, RF energy can also generate active molecules like singlet oxygen that have strong oxidative properties for action on stains and the destruction of cells. 30 This bactericidal action of the RF current, which was evident with the three plaque thicknesses and more significant in the presence of toothpaste, was confirmed by the result of the cell viability analysis with CLSM that showed a significant reduction in the percentage of viable bacteria cells in the biofilm treated with RF when compared with the biofilm not treated with RF energy. This result also accepted our hypothesis that the application of 3-MHz bipolar RF delivered via a power toothbrush RF electrode after biofilm formation would disrupt the bacterial cellular component. The antimicrobial action of the RF indicates that the RF energy acts on multiple targets, bacterial chain formation, and bacterial components. This must undoubtedly have contributed to the reduction in plaque observed with the RF treatments.

Although the present study used the periodontal disease-causing three red complex bacteria ( *P. gingivalis* , *T. denticola* , and *T. forsythia* ) to develop a multispecies dental plaque, it is strongly believed that the ability of the RF energy to kill the bacteria and disintegrate its structural morphology is broad spectrum and not species specific. The result of the present study indicates that the approach of using an RF-utilizing power toothbrush has the potential to contribute toward a better oral hygiene procedure and could be a useful device to eradicate biofilm formation within the oral cavity and, as such, control the two major oral diseases, dental caries and periodontal diseases. Furthermore, considering that calculus is formed by calcification of undisturbed dental plaque by calcium in the saliva, undoubtedly, the destruction of bacteria and their associated biofilm will reduce the rate of calculus formation on tooth surfaces in individuals using the RF-utilizing toothbrush for their routine oral hygiene.

The present study demonstrated that the application of 3-MHz bipolar RF delivered via a power toothbrush RF electrode can disrupt dental plaque morphology and kill its component bacteria cells. The study further demonstrated that the effect of RF energy on plaque was enhanced by the combined application of RF and toothpaste applied as either TS or supernatant of CTS. Furthermore, the effect of RF energy in the present study can be presumed to be the mechanism by which the RF-utilizing toothbrush caused more reductions in plaque and gingivitis as well as extrinsic dental stains in groups that used RF toothbrushes compared with the control group in previously reported studies.

One of the limitations of this study was not comparing the RF-utilizing toothbrush with a non-RF toothbrush. However, we considered the experimental groups that were subjected through the same protocol but not treated with RF energy as adequate control groups for the study objectives. Other limitations were not including a simple thermal camera to show whether or not heating takes place and lack of exhaustive control of confounding factors, such as having more groups with (1) RF electrodes without bristles; (2) nonworking toothbrush without RF; (3) only a vibrating toothbrush, with no RF; and (4) RF generated by a different means. We are presently investigating these factors in our current ongoing study.
